# The impact of COVID-19 on healthcare booking and cancellation patterns: time series analysis of private healthcare service utilisation in Finland

**DOI:** 10.1186/s12913-024-10987-0

**Published:** 2024-04-18

**Authors:** Oskar Niemenoja, Antti-Jussi Ämmälä, Sari Riihijärvi, Paul Lillrank, Petri Bono, Simo Taimela

**Affiliations:** 1 Terveystalo Plc, Helsinki, Finland; 2 Aalto University, Espoo, Finland; 3 University of Helsinki, Helsinki, Finland

**Keywords:** COVID-19, Cov-SARS-2, Digital health, Health service utilisation, Infection epidemiology, Health policy

## Abstract

**Background:**

COVID-19 has had wide-reaching effects on healthcare services beyond the direct treatment of the pandemic. Most current studies have reported changes in realised service usage, but the dynamics of how patients engage with healthcare services are less well understood. We analysed the effects of COVID-19 on healthcare bookings and cancellations for various service channels between January 2020 and July 2021.

**Methods:**

Our data includes 7.3 million bookings, 11.0 million available appointments, and 405.1 thousand cancellations by 900.6 thousand individual patients between the ages of 18 and 65 years. The data were collected from electronic health record data, including laboratory and imaging services as well as inpatient stays, between January 2017 and July 2021. The patients were Finnish private and occupational healthcare customers in the capital region of Finland. We fitted an autoregressive moving average (ARIMA) model on data between 2017 and 2019 to predict the expected numbers of bookings, available appointments, and cancellations, which were compared to observed time series data between 2020 and 2021.

**Results:**

Utilisation of physical, in-person primary care physician appointments decreased by up to 50% during the first 18 months of the pandemic. At the same time, digital care channels experienced a rapid, multi-fold increase in service usage. Simultaneously, the number of bookings for laboratory and imaging services decreased by 50% below the pre-pandemic projections. The number of specialist and hospital service bookings remained at the predicted level during the study period. Cancellations for most health services increased sharply by up to three times the pre-COVID levels during the first weeks of the pandemic but returned to the pre-pandemic levels for the rest of the study period.

**Conclusions:**

The reduction in in-person appointments and the increase in the utilisation of digital services was likely a contributing factor in the decrease of the utilisation of diagnostic and imaging services throughout the study period. Utilisation of specialist care and hospital services were not affected. Cancellations contributed to the changes in service utilisation only during the first weeks of the pandemic.

**Supplementary Information:**

The online version contains supplementary material available at 10.1186/s12913-024-10987-0.

## Background

Previous research has shown that epidemics and pandemics lead to changes in healthcare utilisation patterns. Some studies have indicated a decrease in demand for non-COVID-19 related services, both in primary and emergency care [1, 2]. Previous studies have identified a large initial decrease in health service usage in the early COVID-19 pandemic in the US, the UK, Italy, Finland and other countries [3–8]. A similar effect has been observed in diagnostics and imaging [9]. Similar findings were reported for SARS and Ebola, with an overall reduction of 20% to 30% in health service usage in Taiwan and West Africa, respectively, during these epidemics [10, 11]. At the same time, demand for telemedicine services has increased markedly during the pandemic [12–15]. Cancellations of appointments by the patient or the professional have been suggested to be a notable contributing factor to the decrease in service usage, with some studies noting an increase in cancellation rates during the COVID-19 pandemic [14, 16–18].

To date, most existing literature focuses only on the initial phase of the pandemic during early 2020, where the longer lasting effects of COVID-19 cannot be discerned. Understanding the changes in utilisation rates between digital and physical service channels would benefit from comprehensive data over longer periods of time. Most existing research also approaches the topic via realised service usage, via healthcare spending or the number of reported visits. This omits the behavioural trends on how patients interacted with the services and what effects the pandemic had on booking and cancellation rates.

This study aims to address these gaps in the literature by introducing a more comprehensive view of health service utilisation during the Covid-19 pandemic. Using a novel dataset of Finnish healthcare service booking data spanning the years between January 2017 and July 2021, we analysed how bookings, cancellations and the number of available appointments were affected by the COVID-19 pandemic. These were explored between different digital and physical service production channels. We also established a baseline using data between 2017 and 2019 to calculate the predicted baseline demand for services without the systemic effects of COVID-19.

By shedding light on the impact of the COVID-19 pandemic on health service booking patterns, this study adds to the existing literature on pandemics and healthcare utilisation, demand, and supply. It also offers insight into how the changes in service usage between the digital and physical channels co-affect healthcare delivery. The results of this study may inform healthcare policy and decision-making, particularly in relation to pandemic preparedness and response.

## Methods

### Setting

The Finnish healthcare system is a primarily publicly funded, decentralised system that is comprised of public (publicly paid, publicly or privately provided), private (privately paid, privately provided) and occupational (employer paid, privately provided) healthcare services. Care is typically free or heavily discounted at the point of service for public and occupational health services, whereas private services are funded by the patient or via optional personal health insurance. The data set utilised for the study is from the largest private and occupational healthcare provider in Finland, providing approximately 15% of all outpatient general practice and specialist consultation appointments annually.

The COVID-19 pandemic had a significant impact on the Finnish healthcare system. The first confirmed cases of COVID-19 were reported in Finland in late February 2020, and the number of cases increased rapidly in March and April [19]. To mitigate the spread of the virus, the Finnish government introduced a range of restrictions, including travel restrictions, social distancing measures, and a ban on public gatherings. The government also urged citizens to work from home, avoid unnecessary travel, and practice good hygiene. Healthcare users received recommendations to avoid non-urgent care and favour digital channels where possible. Some Finnish healthcare services were temporarily suspended or scaled down to prioritise COVID-19 patients [16, 20]. The Finnish government declared two states of emergency during the pandemic: at the beginning of the pandemic and at the beginning of 2021. These represent the heaviest regulatory measures to restrict the spread of the pandemic.

### Data

The data set comprised weekly booking data associated with service usage between January 2017 and July 2021. The data set included 7 293 506 bookings, 11 025 216 available appointments and 405 129 cancellations by 900 572 patients. These were considered within distinct service production channels: physical appointments containing in-person outpatient services; digital services containing chat and video; laboratory services; imaging services; and inpatient hospital services. Of these, in-person appointments were further divided into primary care practitioners, including both general and occupational health practitioners, and specialist time types. Furthermore, COVID-19-related diagnostic testing in the laboratory services was separated from traditional laboratory services. The unit sizes and the number of unique patients and appointments of the different groups are presented in the Supplementary Material.

Due to the occupational healthcare focus of the data set, data was limited to bookings by persons between the ages of 18 and 65 years. Different regions in Finland entered different phases of the pandemic at different times [19]. To ensure homogeneity and to limit the effect of regional differences, we limited the study to the capital region of Uusimaa, containing the capital region of Helsinki and neighbouring municipalities with a total population of 1.29 million (29.9% of the Finnish population). The data set was anonymised and did not contain personally identifiable data or demographic information beyond the inclusion criteria by age.

The number of available appointments was calculated as the number of appointments which were available for booking by the public. This represents the total available supply of services during the period. Bookings denote the number of appointments that were ultimately booked. The booking ratio was calculated as the number of bookings divided by the number of available appointments.

Patients may actively disengage from planned care by cancelling already booked appointments. To assess the effect of cancellations on service usage, we included cancellation data in the data set. We calculated both the absolute number of cancellations and the cancellation ratio, which was the weekly number of cancellations divided by the number of bookings. Cancellations by both the patients and the healthcare personnel were included in these numbers.

COVID-19-related testing began in March 2020 and was not present in the data before this date. Digital services existed as a service before 2020, but usage was meagre before the beginning of the pandemic. Hospital services were only conducted in a small number of highly specialised units.

### Statistical methods

We fitted a seasonal autoregressive integrated moving average (ARIMA) model on weekly time series data for bookings, cancellations and available appointments based on data between 2017 and 2019, creating a separate model for each outcome variable. These were used to forecast the point estimates and their confidence intervals (CI) for the expected numbers of respective values in a hypothetical scenario without the effects of COVID-19 between January 1st 2020 and July 15th 2021. These estimates were compared numerically and visually with the observed values to assess the effect of COVID-19 across different service production channels. The models were trained with weekly time series data between 2017 and 2019. The model parameters (*p, d, q*) were calculated so that the Akaike Information Criterion, AIC, was minimised for each model, and the quality of fit was assessed by confirming that the residuals were not correlated using the Ljung-Box portmanteau test with a criterion of *p* > 0.05.

The calculations were performed using the R statistical language (version 4.0.5) and the forecast package [21, 22]. For the predictions, 95% pointwise CIs are presented. Codes are available upon reasonable request from the authors.

## Results

**Fig. 1:**
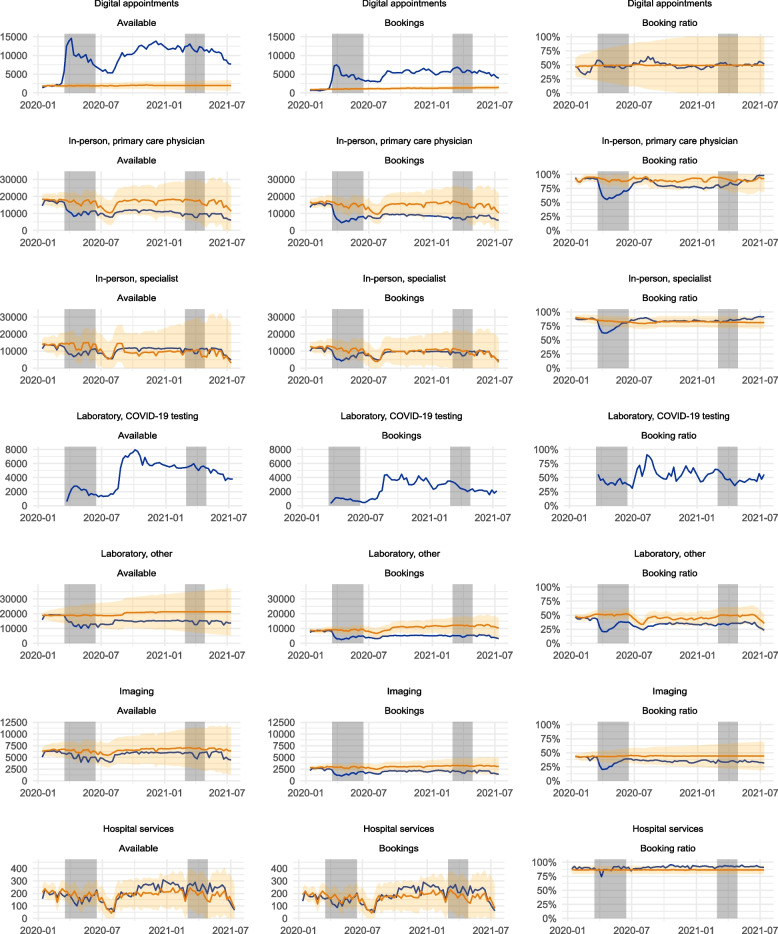
Service supply and bookings in Uusimaa, Finland, between January 2020 and July 2021. The grey areas represent periods of state of emergency declared by the Finnish government and coincide with the heaviest restrictive measures. In-person services are divided into general practitioner and specialist care. Respiratory infection-related tests (COVID-19 tests) are separated from laboratory testing. These were introduced in March 2020, so no prior baseline was calculated. The blue line denotes the observed values, the orange line the predicted values, and the orange ribbon denotes the 95% CI for the predicted values. The predicted values were estimated using time series data from years between 2017 and 2019

Figure 1 depicts the predicted and observed numbers of bookings and available appointments, as well as the ratio between these, during the observation period. Full graphical timelines from 2017 onwards with the estimates, as well as the exact numerical values for the changes in service utilisation reported in the results in tabular format, are presented in the Supplementary Material of this article.

### Digital appointments

Digital appointments saw a significant increase in supply and demand during the pandemic. During the initial onset of the pandemic, the booking volume increased by 368%. This demand persisted through the whole observation period with some fluctuation. The booking ratio remained relatively close the predicted point estimates and within the confidence interval for the estimate during the observation period, and changes in the booking ratio were generally smaller than for the other service channels. This persisted even during periods of high volatility in service demand.

### In-person appointments

During the first period of the state of emergency, primary care physician (PCP) bookings decreased on average by 55%, and specialist bookings decreased by 40%. Booking ratios decreased by an average of 28% and 14% for PCP and specialist care, respectively, during the first state of emergency. PCP bookings remained generally below or at the lower limit of the predicted 95% CI during the observation period, but specialist bookings and time availability recovered to pre-pandemic projections after the first state of emergency. For PCP appointments, the booking ratio generally remained near the lower limit of the 95% CI for the duration of the study, suggesting that supply adjusted to the changes in demand only partially.

### Diagnostics services

Both laboratory and imaging services acted similarly to in-person PCP appointments, with an initial decrease in service usage on average, a 57% decrease in bookings for laboratory and a 48% decrease for imaging services, respectively. The number of bookings for these services remained at these levels for the duration of the study period. Booking ratios stabilised to values between 20 to 30% below the pre-pandemic projections. COVID-19 laboratory testing was introduced as a novel service channel and thus was unique among the observed service production channels, which saw a rapid upscaling both in demand and supply during the pandemic.

### Hospital services

Hospital services were largely unaffected by the pandemic, generally remaining within the 95% CI for the predicted service usage or slightly exceeding those.

### Cancellations

**Fig. 2:**
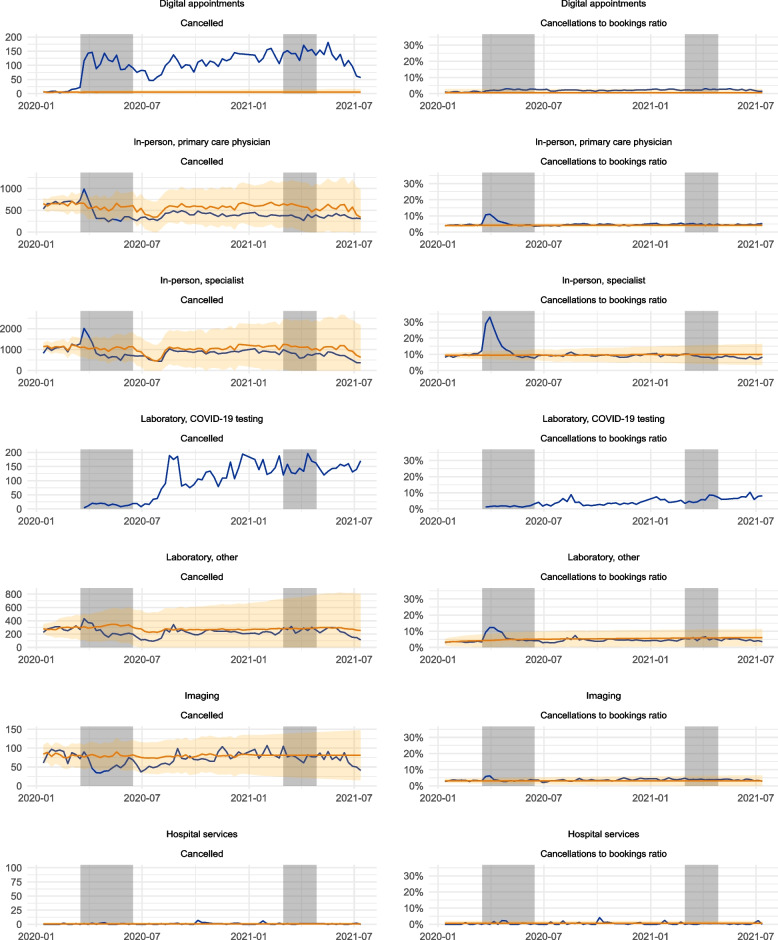
Cancellations and the ratio of cancellations to bookings in Uusimaa, Finland, between January 2020 and July 2021. The grey areas represent periods of state of emergency declared by the Finnish government, coinciding with the heaviest regulatory measures. Only cancellations by the patient or the physician were considered. The blue line denotes the observed values, the orange line the predicted values, and the orange ribbon denotes the 95% CI for the predicted values. The predicted values were estimated using time series data from years between 2017 and 2019

Figure 2 depicts cancellations by patients or medical personnel during the observation period between January 2020 and July 2021. Both the absolute number of cancellations, as well as the ratio of cancellations to bookings, are depicted. Here, digital and in-person appointments as well as imaging and non-COVID-19 laboratory services experience a cancellation shock during the initial phase of the pandemic, after which the values generally return to values within the 95% CI. COVID-19 laboratory testing services saw moderate increase over the pandemic, but cancellations of hospital services were generally not affected by the pandemic. In-person appointment cancellation ratios peaked at around three times the predicted rate, to 11% and 33% of bookings, for PCP and specialist appointments respectively. Both laboratory and imaging diagnostics cancellation ratios displayed similar behaviour, with an increase from 4 to 12% during the early pandemic for laboratory services and 3% to 6% for imaging services, respectively. For digital services and hospital services, this pattern of higher-than-predicted rates of cancellations to bookings is not noted during the early pandemic.

## Discussion

Visits at primary care physicians, imaging and laboratory services saw a notable decrease in service usage that was especially prominent in the early pandemic and persisted through the observation period. However, the relatively stable number of specialist appointments and hospital services during the study period suggest that specialised care continued relatively unaffected during the pandemic. The shift in the utilisation of service channels, with the simultaneous increase in digital service utilisation and the decrease within physical service channels, may have resulted in an overall decrease in laboratory tests and imaging services.

Potential explanations to forgo diagnostic services in digital channels may include difficulty of access, where patients not already located at the care premises are thought to be inconvenienced by a separate visit to a laboratory; perceived willingness to pay the cost of the service, as digital appointments are typically cheaper than in-person appointments; and a decrease in “just in case” diagnostics, where referrals are prescribed in cases where conventional diagnostics by the doctor would suffice. Digital service physicians may not be familiar with the location and availability of diagnostic and imaging services near the location of the patient. The lower cost and accessibility of digital services may also promote health service usage for milder conditions than traditionally would be treated with an in-person visit, modifying patient mix, and lessening the need for laboratory and imaging services.

Beyond diagnostics, laboratory services include large-scale testing such as employment-related health screening, testing for sexually transmitted diseases, controls for chronic conditions, and seasonal influenza. All of these may have seen an organic decrease due to COVID-19, even without changes in referral patterns. Imaging includes, for example, diagnostics for accidents and respiratory system-related conditions, which may have seen a similar reduction. Controlled downscaling of non-emergency appointments and activity to preserve healthcare personnel for COVID-19 testing and tracing was a central strategy of the Finnish pandemic response [16, 20]. These may be limiting factors for the available supply across all service channels. The most severe cases of probable COVID-19 were typically transferred to public university hospitals, so the number of imaging related to severe COVID-19-related complications is likely absent from this data set. One exception was the COVID-19 testing services, which were heavily used during the pandemic. The COVID-19 related testing is likely most affected by fluctuations in external demand, where companies or municipalities were major payers for large-scale testing. Changes are more likely to reflect population-level rather than personal-level demand for services.

The changes in bookings reflect passive disengagement from health services, where previously requested care was omitted or not made available in the first place. Cancellations, on the other hand, reflect the patients’ or professionals’ actions to actively disengage from the health services. We can observe a major spike in cancellations immediately following the onset of the pandemic, with a threefold increase in cancellation rates for in-person appointments. In-person specialist cancellations peaked at over 30% during the first weeks of the pandemic. However, this period of rapid disengagement lasted a relatively short time, with the services returning to pre-pandemic cancellation rates for the remainder of the pandemic. Cancellations have, at times, been portrayed as a reason for a major decrease in service usage [17]. Our results suggest that this was a contributing factor mainly during the first weeks of the pandemic, after which disengagement from the services was passive.

The popular shift to digital services has been largely documented in previous studies [12–15]. We add to the existing literature by observing that the booking ratios remained relatively stable during the pandemic, even during periods of rapid changes in service demand. This suggests that digital services can scale supply to match rapid changes in demand to provide timely access to healthcare during pandemics. Digital services can, therefore, act as a rapidly scalable buffer to regulate access to care during pandemics.

### Strengths and limitations

We had access to a large and comprehensive health register spanning an 18-month period from the onset of the COVID-19 pandemic, comprising a homogenous population in the same geographical region. With the introduction of booking and cancellation data, our study extends the earlier results on the changes in service utilisation rates [5, 6, 9, 14, 23, 24]. High-quality booking data is not typically available for time series analysis, which usually focuses on realised service usage. We also had access to data from previous periods, enabling us to create credible estimates with seasonal and yearly trends for the nominal usage of health services without the impact of the COVID-19 pandemic.

A limitation of the study is that the population is comprised of private healthcare customers, which may not represent the population at large. Patients utilising other service providers are not represented in the data set. The most difficult COVID-19 cases, including hospitalisations, were treated within the public health services and are not present in the data set. As predictive estimates are less accurate over longer periods of time, the paper included a relatively short period of 18 months.

## Conclusions

Digital services can act as a rapidly scalable buffer to regulate access to care during pandemics. However, the change in the ways patients interact with healthcare services by shifting service usage to digital channels was likely a contributing factor to the decreased utilisation of diagnostic and imaging services during the study period. Specialist care and hospital services were not similarly affected by this shift. The possible lasting effects of the decreased diagnostic activity are yet unknown, and the effects may appear only long after the acute phases of the pandemic are over. Future scholars will hopefully further explore how healthcare systems at large are affected during pandemics. Better understanding of these system-wide effects is key to managing the secondary effects of any future external shocks to healthcare systems.

### Supplementary Information

**Supplementary Material 1. **

## Data Availability

Data requests are managed through the Finnish Social and Health Data Permit Authority Findata. The codes are available from the author upon reasonable request.
