# Supplementary material for: The impact of COVID-19 on healthcare booking and cancellation patterns: time series analysis of private healthcare service utilisation in Finland
Source: BMC Health Serv Res. 2024 Apr 18;24:483. doi: 10.1186/s12913-024-10987-0 (PMC11027366; doi:10.1186/s12913-024-10987-0)
Supplement: Supplementary file 1 — Supplementary Material 1. [file 12913_2024_10987_MOESM1_ESM.pdf]

Supplementary material

Supplementary material A

Table A: Descriptive statistics for the data set. The number of individual persons and the number of individual appointments are reported for each service channel and time type, where applicable. Model refers to the pre-2020 data used to train the baseline model, whereas Observation refers to the observed post-2020 data. Total contains the combined number of unique patients and appointments across the whole study period. For the Available bookings, only the number of appointments is reported, as these represent the total supply of possible appointments. For Bookings and Cancellations, the number of unique persons is also reported. Covid-19 testing began only after the onset of the pandemic, and no pre-pandemic model data exists.

	Available	Bookings		Cancellations	
	Appointments	Unique persons	Appointments	Unique persons	Appointments
Overall					
Baseline model *	6 783 229	675 326	4 872 557	147 083	269 449
Observation **	4 241 987	549 656	2 420 949	85 302	135 680
Total	11 025 216	900 572	7 293 506	203 950	405 129
In-person, Primary Care Physician					
Baseline model *	2 352 916	493 989	2 121 098	62 463	86 861
Observation **	843 381	283 654	684 659	26 225	32 315
Total	3 196 297	589 691	2 805 757	82 929	119 176
In-person, Specialist					
Baseline model *	2 181 430	449 372	1 751 716	83 171	134 725
Observation **	798 205	242 853	666 299	42 898	65 105
Total	2 979 635	537 077	2 418 015	114 364	199 830
Digital appointments					
Baseline model *	104 157	35 914	43 310	176	186
Observation **	723 327	189 370	358 271	6 247	7 826
Total	827 484	211 855	401 581	6 414	8 012

Laboratory					
Baseline model *	1 363 505	281 541	663 393	30 645	38 069
Observation **	1 136 148	192 099	391 877	15 040	18 052
Total	2 499 653	370 021	1 055 270	43 910	56 121
Laboratory, COVID-19 testing					
Baseline model *	*NA*	*NA*	*NA*	*NA*	*NA*
Observation **	295 200	98 081	155 324	6 006	6 855
Total	295 200	98 081	155 324	6 006	6 855
Imaging					
Baseline model *	756 640	161 184	271 837	8 085	9 480
Observation **	430 230	100 154	150 387	4 664	5 460
Total	1 186 870	223 669	422 224	12 554	14 940
Hospital services					
Baseline model *	24 581	16 006	21 203	104	128
Observation **	15 496	8 842	14 132	56	67
Total	40 077	23 956	35 335	160	195

* 1.1.2017 - 31.12.2019

** 1.1.2020 – 1.7.2021

Supplementary material B

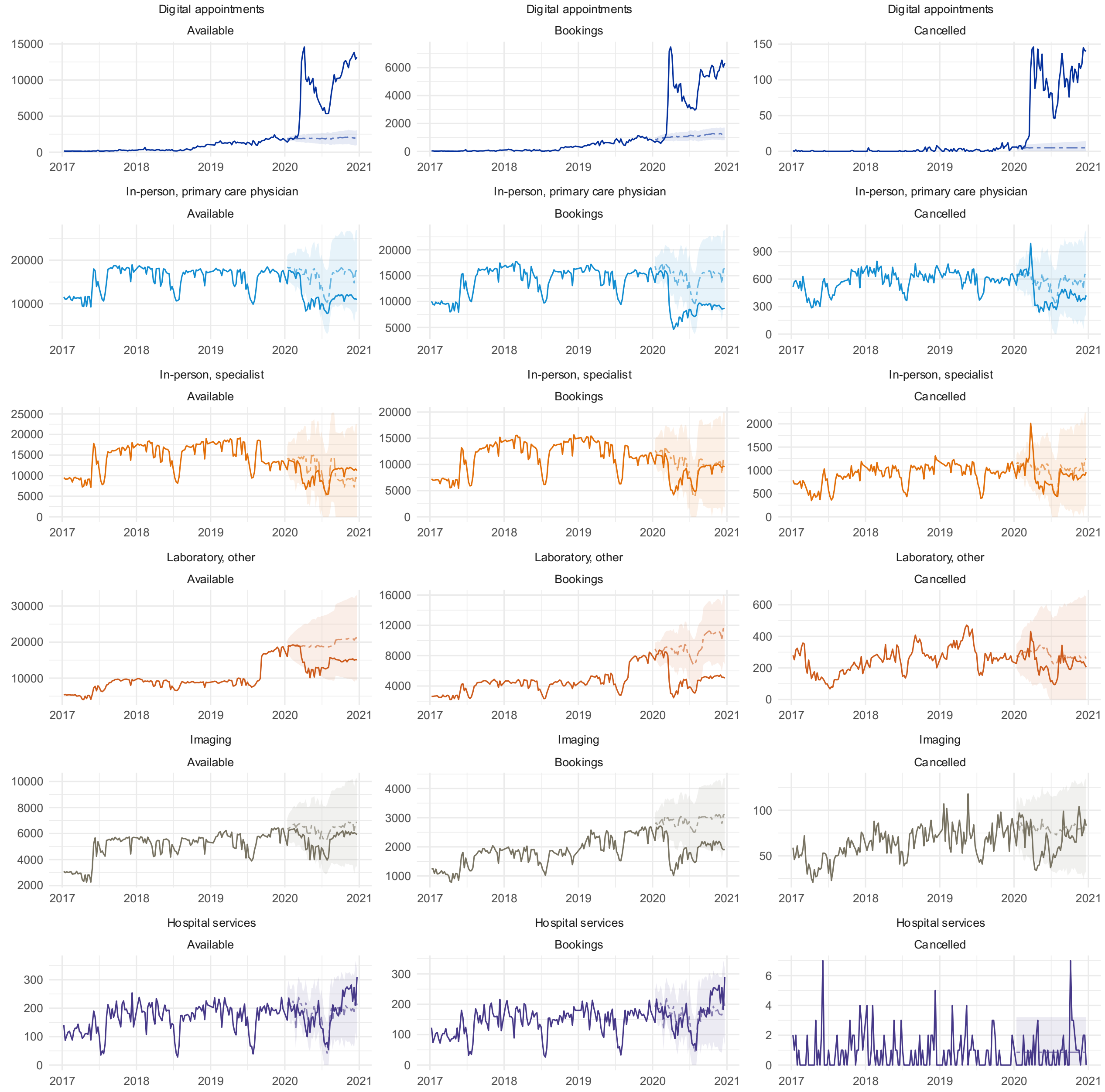

Figure B: Time series for the individual service production channels across bookings, cancellations, and the numbers of available appointments. The solid line represents observed values, while the dotted line represents estimated activity without the effects of COVID-19. The ribbons, visible from

2020 onwards, refer to the 95% CI of the values. The estimate is built on the weekly values between 2017 and 2019.

Supplementary material C

Table C: The difference between observed and estimated service usage within the different service production channels. The ratios represent total observed service usage divided by the expected, estimated service usage across five periods during the pandemic. The periods highlighted with a grey colour correspond to the states of emergencies in Finland. Positive values correspond to periods where the observed values were greater than the estimate, whereas negative values correspond to periods where the observed values were smaller than the estimate.

	Period	2020-01-01 - 2020-03-16	2020-03-17 - 2020-06-15	2020-06-16 - 2021-02-28	2021-03-01 - 2021-04-27	2021-04-28 - 2021-06-28	Overall
Available	Digital appointments	22,1 %	433,4 %	420,0 %	499,7 %	429,2 %	381,8 %
	In-person, primary care physician	-6,9 %	-38,2 %	-34,2 %	-46,6 %	-44,4 %	-33,7 %
	In-person, specialist	-6,6 %	-33,2 %	11,6 %	11,2 %	17,3 %	-0,8 %
	Laboratory	-0,3 %	-32,9 %	-28,0 %	-31,1 %	-32,0 %	-26,2 %
	Imaging	-7,4 %	-20,4 %	-13,4 %	-17,1 %	-15,4 %	-14,5 %
	Hospital services	-6,6 %	-12,9 %	14,2 %	24,6 %	25,5 %	9,1 %
Bookings	Digital appointments	11,2 %	368,1 %	317,9 %	352,9 %	283,3 %	293,0 %
	In-person, primary care physician	-8,0 %	-55,4 %	-41,3 %	-52,5 %	-43,7 %	-40,5 %
	In-person, specialist	-9,3 %	-40,4 %	-5,2 %	-11,8 %	4,2 %	-12,4 %
	Laboratory	-5,2 %	-57,4 %	-52,7 %	-57,8 %	-58,0 %	-49,5 %
	Imaging	-12,4 %	-47,8 %	-33,4 %	-38,9 %	-38,6 %	-34,5 %
	Hospital services	-7,3 %	-14,9 %	15,5 %	25,1 %	25,0 %	9,4 %
Cancelled	Digital appointments	93,0 %	2179,5 %	2061,7 %	2828,7 %	2537,9 %	1965,8 %
	In-person, primary care physician	2,9 %	-29,2 %	-30,3 %	-39,3 %	-35,6 %	-26,9 %
	In-person, specialist	-3,8 %	-13,6 %	-16,7 %	-30,6 %	-31,8 %	-18,0 %
	Laboratory	-2,4 %	-22,2 %	-21,1 %	-4,4 %	-17,2 %	-16,3 %
	Imaging	1,1 %	-31,8 %	-8,3 %	-3,2 %	-9,0 %	-10,6 %
	Hospital services	-64,8 %	-0,8 %	37,9 %	-21,9 %	-34,9 %	1,6 %
